# Feeding of Marine Zooplankton on Microplastic Fibers

**DOI:** 10.1007/s00244-022-00948-1

**Published:** 2022-07-28

**Authors:** Marion Köster, Gustav-Adolf Paffenhöfer

**Affiliations:** 1 Institut für Mikrobiologie, Universität Greifswald, Greifswald, Germany; 2 Skidaway Institute of Oceanography, University of Georgia, Savannah, GA 31411 USA

## Abstract

The goal of our study was to examine the effects of low abundances of nylon fibers on feeding rates of calanoid copepods (Crustacea, Copepoda) and doliolids (Tunicata, Thaliacea) in the presence of diatoms at near environmental concentration levels. In addition, we examined microscopically the fecal pellets produced by copepods and doliolids in the presence of fibers. Adult females of the calanoid *Eucalanus pileatus* and early gonozooids of *Dolioletta gegenbauri* (both of similar dry weight) cleared the diatom *Rhizosolenia alata* at similar rates. Nylon fibers were cleared at higher rates by *Dolioletta gegenbauri* compared to *Eucalanus pileatus*. Examination of fecal pellets revealed that copepods and doliolids could ingest the about 300 µm long fibers. The latter also ingested the occasionally occurring fibers of > 1 mm length. It appears that in seawater fiber abundances of about seven fibers ml^−1^ did not have a negative effect on feeding of either *E. pileatus* or *D. gegenbauri*. As doliolids and copepods remove plastic fibers from seawater by packing them into their pellets, they might play a role in the reduction of microplastic pollution and the microplastic transfer from the water column to the seafloor. Calanoid copepods may limit ingesting fibers by not perceiving them, as compared to doliolids which do not seem to be able to avoid ingesting them.

The occurrence of microplastic particles in the ocean has been observed for decades (e.g., Andrady 2011). Initially, they were defined as smaller than 5 mm (Arthur et al. 2009). They occurred in the open ocean at high abundances in the range of 1–10 mm when collected with a 200 µm mesh net (Cozar et al. 2014). Those authors pointed out that our knowledge on particles < 1 mm is limited. Quantification of smaller sizes (> 0.7 µm) was achieved by Di Mauro et al. (2017) collecting with Niskin bottles and filtering on glass fiber filters. Their microscopic filter examination provided concentrations of microplastics of > 100 L^−1^. Recently, Brandon et al. (2020) quantified < 333 µm microplastic particles in surface seawater samples and salp gut contents, using a new microscopic technique, and also those of > 333 µm length in the water column. Their results, from nearshore to offshore Pacific Ocean, revealed concentrations of microplastic particles from near 4000 to about 15,000 L^−1^, mainly short fibers of an average of 60 µm length. Those small fibers are of a size which could be readily ingested by juveniles and adults of marine planktonic copepods.

There have been quite a few studies on the influence of microplastics on aquatic invertebrates, especially zooplankton (e.g., Cole et al. 2015; Coppock et al. 2019; Botterell et al. 2019). Among the zooplankton are the planktonic copepods which are the most abundant metazoans on our planet (Fryer 1986). Early it was shown that various species of planktonic copepods were feeding on polystyrene beads, but so did also doliolids (Tunicata, Thaliacea) and euphausiids (Cole et al. 2013). Later we learned that polystyrene beads led to a decrease of feeding on phytoplankton (Cole et al. 2015, Table 1). Such polystyrene beads would also negatively affect feeding, growth and oxygen consumption of doliolids (Paffenhöfer and Köster 2020, Table 1). Recently Coppock et al. (2019, Table 1) offered nylon fibers and fragments (10 µm diameter, 40 µm length) to the planktonic copepod *Calanus helgolandicus* resulting in a substantial ingestion decrease of chain-forming diatoms and also of unicellular microalgae. Cole et al. (2019) recorded a 40% reduction of phytoplankton ingestion by *C. finmarchicus* when fibers were present (Table 1). The concentration of beads or fibers ranged from 37 to 100 ml^−1^ (Table 1), the accompanying phytoplankton concentrations from 67 to 327 µg C L^−1^ (Table 1). As to the effects of microplastic particles on marine zooplankton, a review by Botterell et al. (2019) indicated that negative effects were reported in 45% of the studies, while in 14% (three studies) no effects were found.

**Table 1:** The effects of polystyrene beads and fibers on the feeding of marine zooplankton

	Food type	Food/micro-plastic concentration	Volume of vials (L)	Feeders per bottle	Tempe-rature (°C)	Duration (h)	Results	References
*Calanus helgolandicus*	Polystyrene beads 20 µm diameter	75 mL^−1^	0.617	5	11	24	Feeder *C. helgolandicus* ingested 11% fewer algal cells when exposed to beads and *T. weissflogii*, no effects on survival and egg production	Cole et al. (2015)
	*Thalassiosira weissflogii*	250 µg C L^−1^						
*Dolioletta gegenbauri*	Polystyrene beads 20 µm diameter	37 mL^−1^	0.96–1.9	9–11	20	23 two time-series	Feeding rates on phytoplankton decreased as did rates on growth and oxygen consumption	Paffenhöfer and Köster (2020)
	Mixture of *T. weissflogii* and *I. galbana*	67 µg C L^−1^						
	Offering beads and phytoplankton together							
*Calanus helgolandicus* females	Nylon fibers 10 µm diameter, 40 µm length	100 mL^−1^	0.615	5 adult females	11	24	Reduction of feeding rates on phytoplankton in the presence of fibers	Coppock et al. (2019)
	Mixture of *Dunaliella* sp., *Prorocentrum* sp., *Thalassiosira rotula*	120 µg C L^−1^					Volume swept clear on fibers was 10 ml female^−1^ d^−1^	
							Volume swept clear on phytoplankton was 22–27 ml female^−1^ d^−1^	
*Calanus finmarchicus* copepodid V	Nylon fibers 10 µm diameter, 30 µm length	47 mL^−1^	1.15	10	8.7	4 days	40% reduction of phytoplankton ingestion in the presence of fibers	Cole et al. (2019)
	Mixture of *Dunaliella* sp., *T. rotula*, *S. trochoidea*	327 µg C L^−1^						

Environmental observations revealed that fibers often predominate as microplastics in the ocean (e.g., Cole et al. 2011; Desforges et al.^2014^). This led us to our present study, offering nylon fibers to two marine subtropical zooplankton species, the calanoid copepod *Eucalanus pileatus* (Crustacea, Copepoda)*,* and the doliolid *Dolioletta gegenbauri* (Tunicata, Thaliacea)*. D. gegenbauri* has been encountered in high abundances (> 1000 m^−3^) on the US southeastern shelf since April 1975 (Atkinson et al. 1978). It has been observed colonizing much of the SE shelf (Paffenhöfer et al. 1987); and has occurred at temperatures from near 14 to 26 °C. *Eucalanus pileatus* has been found throughout much of the year on the SE shelf, usually at >15 °C (e.g., Bowman 1971), and when phytoplankton is abundant like during intrusions of Gulf Stream upwellings (e.g., Paffenhöfer et al. 1984). This species is also abundant in other subtropical upwellings like off Cabo Frio, Brazil (Valentin and Monteiro-Ribas 1993). The chain-forming diatom *Rhizosolenia alata* is abundantly encountered in intrusions, and is readily ingested by *E. pileatus* (e.g., Paffenhöfer and Van Sant 1985) and *D. gegenbauri* (e.g., Paffenhöfer and Köster 2005, and references therein). *D.* gegenbauri occurs circumglobally on subtropical continental shelves and adjacent waters (e.g., Deevey 1952; Monteiro et al. 1975; Mackas et al. 1991; Paffenhöfer et al. 1995; Deibel 1998; Nakamura 1998; Takahashi et al. 2015). Studies of feeding of doliolids on plastic particles so far were limited to Tebeau and Madin (1994) who did not observe negative effects; and by Paffenhöfer and Köster (2020) who reported on negative effects of polystyrene beads on feeding, growth and oxygen consumption.

Fecal pellets of both copepods and doliolids can contribute significantly to particulate matter (Paffenhöfer and Knowles 1979; Patonai et al. 2011). The actual significance of fecal pellets to ocean biochemical processes has been evaluated by Turner (2002, 2015).

A short yet critical evaluation of microplastic abundances in different parts of the ocean versus their concentrations in experimental studies led to the conclusion to suggest to conduct future studies on the effects of such microplastics on organisms approaching environmental microplastic abundances (Lenz et al. 2016). This suggestion resulted in our plan to offer nylon microfibers at low numerical concentrations, and close to environmental dimensions (e.g., Desforges et al. 2014), together with phytoplankton to compare potential fiber effects on a common calanoid copepod and an often co-occurring doliolid species.

We hypothesize (i) that nylon fibers at their low numerical concentration would not affect feeding rates of calanoid copepods and doliolids at near average environmental diatom abundances and (ii) that fecal pellet content of calanoids and doliolids would differ due to differences of feeding behavior when fibers and diatoms are offered simultaneously.

## Material and Methods

### Zooplankton Collection

The zooplankton species *Eucalanus pileatus* and *Dolioletta gegenbauri* were collected on a cruise on 29 January 2020 near the 40 m isobath on the US southeastern shelf at temperatures of 18 °C. The zooplankton were collected with a net of 0.5 m mouth diameter, 200 µm mesh size and a 4-L codend, being towed at near 0.5 m s^−1^ from near surface to near bottom to the surface. Directly after collection, doliolids and copepods were placed into 3.8 L glass jars which were then fastened to a plankton wheel moving at near 0.5 r.p.m. in an on-board laboratory adjusted to 20–21 °C. At the Skidaway Institute of Oceanography, those jars were placed on a plankton wheel in an environmental room at 20 °C and a light–dark cycle of 12 h:12 h. Both zooplankton species were offered concentrations of the flagellates *Isochrysis galbana, Rhodomonas* sp. and the diatoms *Thalassiosira weissflogii* and *Rhizosolenia alata* at average levels of near 40–60 µg C L^−1^.

### Culture Conditions

To obtain animals for our experiments copepods and doliolids had to be cultured under controlled conditions. Several females of *Eucalanus pileatus* were placed in 3.8 L jars on a rotating wheel being offered *T. weissflogii* and *R. alata* at an average level of about 50 µg C L^−1^, and reproduced readily nauplii that were grown to adult females offering the above-mentioned mixture. To obtain gonozooids of *D. gegenbauri* to start our experiments, we cultured this species in our environmental laboratory at 20 °C on a plankton wheel through its life cycle (see Paffenhöfer and Gibson, 1999, for details), offering the previously mentioned phytoplankton species at total average concentrations of 40–60 µg C L^−1^. Concentrations of the different food organisms were quantified daily using a Beckman Coulter Multisizer IV with a 140 µm orifice diameter tubing. The doliolids were cultured in seawater of 36.0%o (parts per thousand) salinity; this water was partly renewed daily removing most of the accumulated fecal pellets, following cultivating details from Paffenhöfer and Gibson (1999).

### Preparation of Microfibers

We selected nylon fibers for our experiments since they are among the most abundant microplastic particles in nature (Cole 2016). The fibers were prepared for these experiments following the protocol developed by Cole (2016). Transparent nylon fibers (polyamide 6,6) of 10 µm width (Goodfellow GmbH, Hamburg, Germany) were aligned, embedded in a water-soluble freezing solution (Neg 50™ Thermo Scientific, UK), and frozen at − 70 °C for at least 1 h. The frozen gel block containing the aligned fibers had a length of 12 cm and was cut with a scalpel into gel blocks with a length of 1 cm. After eight (2 × 4) of those 1 cm long gel blocks were 90°-oriented to the surface of an aluminum holder and closely placed together to a large block, they were embedded in an additional freezing solution and frozen at − 70 °C for 1 h. The frozen gel block containing the fibers was sliced with a stainless steel microtome knife (5° angle) into about 300 µm thick sections in a Bright cryostat microtome (Huntington/Cambridgeshire, UK) at − 25 °C. Frozen sections were collected and thawed in ultrapure water heated at 60 °C to release the fibers. Then, the fibers were filtered on polycarbonate filters (pore size 5 µm, diameter 47 mm) and washed with ultrapure water. Fiber-loaded filters were stored at − 20 °C until experimental use. We had decided on a nominal fiber length of 300 µm because Desforges et al. (2014), using as their smallest collection mesh 64 µm, mentioned that the most abundant size fraction of microplastic particles was that of 100–500 µm (fibers and plastic fragments).

### Microfiber Counts and Sizes

Before experimental start, each fiber-containing filter was inspected for air contamination (e.g., fibers from other sources) and an even distribution of the fibers on the filter surface under a LEICA MZ 12 stereomicroscope (Leica Microsystems, Wetzlar, Germany). Filters that contained foreign fibers and/or revealed an uneven distribution of fibers were not used. For the determination of the number of 300 µm long fibers per filter, 20 microphotographs per filter were taken at 40 fold magnification with a digital 10 MP camera (ISH 1000; Tuscen Photonics, Fuzhou, China) connected to the stereomicroscope. Using the software TCapture (version 5.1), fibers were documented and counted in 10 to 20 randomly chosen 6 mm^2^ areas on the filter surface (corresponding to 4 to 7% of the total effective filtration area). The total number of fibers per filter varied between 12,840 and 17,558. For experimental use, the fiber-loaded filters were cut into halves; then, fibers were cautiously washed off and transferred to one Liter of filtered experimental seawater. Counting microfibers on polycarbonate filters allowed us to adjust roughly the number of added fibers at the beginning of each experiment. Exact fiber counts at the beginning and end of each experiment were obtained from counting fibers in settling chambers by inverse microscopy. The concentrations of fibers chosen for experiments were near 5.7–9.0 fibers ml^−1^ in an attempt to approach natural abundances of microplastics in the ocean (Lenz et al. 2016).

**Fig. 1:**
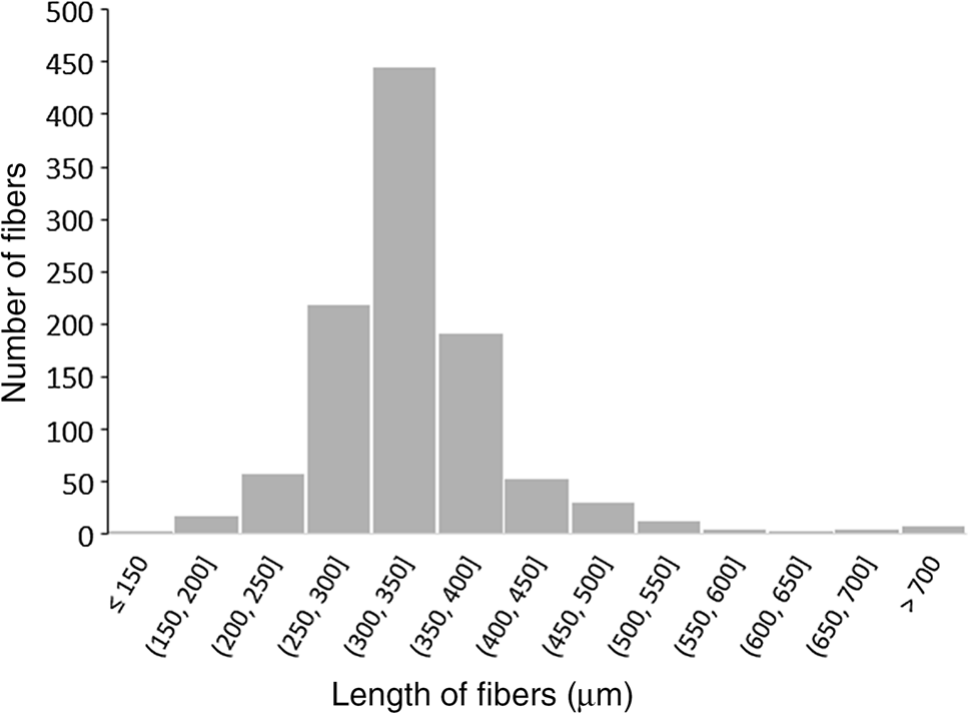
Size distribution of nylon fibers that were sectioned with a microtome adjusted to a nominal length of 300 µm. Fibers obtained from thin sections were filtered on the surface of polycarbonate filters (diameter of 47 mm). The lengths of 1060 nylon fibers were measured using the software MikroCamLab II. The average length of fibers was 336 µm ± 91 Standard Deviation. Note that there are a very few fibers with multiple nominal lengths

The microphotographs (at 40-fold magnification) were also used to determine the length and the size distribution of the microfibers obtained by adjusting the thickness of the microtome sections to 300 µm. The exact length of approximately 200 fibers was determined on each of five polycarbonate filters (diameter of 47 mm) using the software MikroCamLab II. The fiber length distribution of 1060 microtome-cut fibers (Fig. 1) shows that 42% of the fibers had a length of ≥ 300 to < 350 µm, 21% and 18% of fibers were in the size classes ≥ 250 to < 300 µm, and ≥ 350 to < 400 µm, respectively. The average length of the fibers was 336 µm (standard deviation 91). We observed that we had not always succeeded to produce fibers with the nominal length of 300 µm. Some fibers escaped sectioning and had double or triple length of the adjusted length. Fibers with multiple size (> 600 µm) contributed less than about 2% of the total number of cut fibers.

### Experimental Design

All experiments were carried out at 20 °C on a plankton wheel running at 0.3 r.p.m. at a 12 h/12 h light–dark cycle. We offered the elongated diatom *R. alata* and nylon fibers together at similar particle concentrations each ranging on average between 5.7 and 9.0 ml^−1^. *R. alata* concentrations resembled those found in situ (Paffenhöfer, unpubl. results). The diatom cells were of 35–38 µm width and an average length of 250 µm. The fibers were of 10 µm width and on average 300 µm long, thus being of similar length as the diatoms. Our initial experiment with *E. pileatus* was run for 18 h, and the remaining five for 6.0 to 6.1 h. All experiments were run in 960 ml screw cap bottles with five young females, i.e., just molted adult females. All five experiments with *D. gegenbauri* were run for 6.0 to 6.1 h with five gonozooids of an average length of 4.5 to 4.9 mm. Those sizes were chosen as their average biomass carbon was 16–18 µg and similar to that of young females of *E.* *pileatus.* Controls were run in 960 ml jars to quantify the growth rate of and the feeding rates on *R. alata.* We decided not to offer the fibers by themselves as there are always living phytoplankton particles in the epipelagic ocean. Chlorophyll a levels in surface waters of the open ocean are always near or above 0.04 µg L^−1^. Phytoplankton and fiber concentrations were quantified by inverted microscope counts. For each experiment, we counted fibers and *R. alata* cells in three settling chambers of 25 ml each at the start and end of each experiment. In parallel we ran control feeding experiments in 960 ml jars at same feeder and *R. alata* concentrations (five *E. pileatus* females, or five *D. gegenbauri* gonozooids of 4.5 to 4.9 mm length) over same feeding periods, but no fibers added!

Feeding/clearance/ingestion rates were calculated according to Frost (1972) who described all calculations in detail. The clearance rate is the amount of water (ml) swept clear by an individual zooplankter per hour or day; the ingestion rate is the amount of particles ingested by an individual zooplankter per hour or day. The lengths and stages of the experimental animals were determined at the beginning and end of each experiment. The length values of doliolid gonozooids were transformed to biomass carbon values using the equation weight (µg C) = 0.4643 length (mm)^2.3119^ (Gibson and Paffenhöfer 2000). No copepods or doliolids died during our experiments. All escaped well at the start and end of each experiment.

### Pellet-Associated Microfibers

Microphotographs of fecal pellets released by *E. pileatus* and *D. gegenbauri* were taken with a digital camera (ISH 1000, Tuscen Photonics, Fuzhou, China) connected to an inverted microscope DIAVERT (Ernst Leitz GmbH, Wetzlar, Germany). All pellets detected in settling chambers were documented at tenfold magnification. The number of fibers in pellets was determined on selected microphotographs. Length and width of fecal pellets were measured using an microscope calibration slide stage micrometer (0.01 mm = DIV). The software TCapture version 5.1 was used for photodocumentation.

### Statistics

Statistical analyses were made according to Zar (1974) applying the Kruskal–Wallis Test. This is a nonparametric single factor analysis of variance by ranks for *K* ≥ 2 independent samples (Conover 1980).

## Results

### Particle Abundances and Feeding Rates

**Fig. 2:**
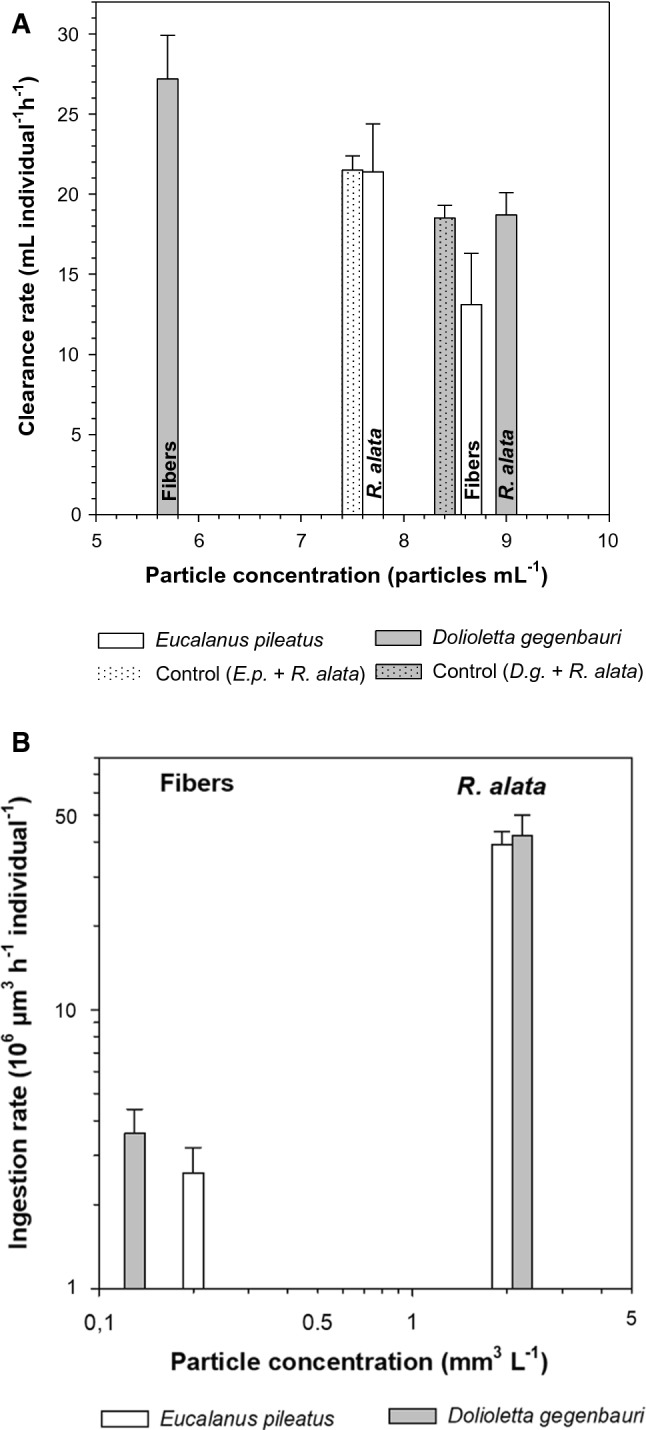
**A** Mean clearance rates of *Eucalanus pileatus* and *Dolioletta gegenbauri* feeding simultaneously on *Rhizosolenia alata* and nylon fibers at 20 °C, and feeding only on *R. alata* (controls). Error bars present ± one Standard Error. **B** Ingestion rates of *Eucalanus pileatus* and *Dolioletta gegenbauri* feeding simultaneously on *Rhizosolenia alata* and nylon fibers at 20 °C

As fibers are considered the dominant microplastics in subsurface waters of the northeast Pacific Ocean (Desforges et al. 2014), we chose them as microplastics representatives. While the average length of the *R. alata* cells at 250 µm was close to that of the fibers at 300 µm, the latter’s width at 10 µm was much lower than that of *R. alata* at 35–38 µm. The average volume of a *R. alata* cell was 0.250 × 10^6^ µm^3^ and that of a fiber 0.235 × 10^5^ µm^3^. The copepod *E. pileatus* readily consumed *R. alata* cells and fibers (Fig. 2A, B). Average experimental concentrations of *R. alata* were 7.71 ± 0.83 Standard Error SE cells ml^−1^, corresponding to a biomass volume of 1.930 × 10^6^ µm^3^ ml^−1^, or a biomass carbon of 28.9 µg C L^−1^. Concentrations and volume of fibers amounted to 8.66 ± 0.39 SE fibers ml^−1^ and a volume of 0.204 × 10^6^ µm^3^ ml^−1^, respectively (Fig. 2 A). The average clearance rate on *R. alata* was at 21.4 ml copepod^−1^ h^−1^, (SE in Fig. 2 A as also for future clearance rates) significantly higher than that on fibers at 13.1 ml copepod^−1^ h^−1^ (Kruskal–Wallis test, *p* < 0.05). It was not possible to count the number of pellets produced by the copepods because they had attacked and damaged numerous pellets. The average clearance rate on *R. alata* in the presence of fibers (21.4 ml female ^−1^ h^−1^) did not differ significantly from that of the control (no fibers, 21.5 ml copepod ^−1^ h^−1^, Kruskal–Wallis test, *p* > 0.05, Fig. 2A).

Gonozooids of *Dolioletta gegenbauri* were feeding on *Rhizosolenia alata* at an average of 8.95 ± 0.91 (SE) cells ml^−1^ (corresponding to 2.20 × 10^6^ µm^3^ ml^−1^or 33.6 µg C L^−1^) and at 5.71 ± 1.65 fibers ml^−1^ (corresponding to 0.134 × 10^6^ µm^3^ ml^−1^; Fig. 2A). The clearance rates on fibers were at 27.2 ml gonozooid^−1^ h^−1^ significantly higher than on *R. alata* at 18.7 ml gonozooid^−1^ h^−1^ (Kruskal–Wallis test, *p* < 0.05). The average clearance rate of a gonozooid of *D. gegenbauri* on *R. alata* in the presence of fibers did not differ significantly from the clearance rate of the controls, i.e., when only *R. alata* was offered (Kruskal–Wallis test, *p* > 0.05, Fig. 2A). Ingestion rates were compared in relation to particle concentration (Fig. 2B). The different particle volumes of fibers and diatom cells resulted in vastly different ingestion rates (Fig. 2B) which were about one order of magnitude higher on *R. alata* than on the fibers. While *D. gegenbauri* ingested about 10% more *R. alata* than *E. pileatus* (partly due to slightly higher *R. alata* abundance), the ingestion rate on fibers by numbers was 31% higher for *D. gegenbauri* than for *E. pileatus*. Enhanced fiber ingestion rates of *D.* *gegenbauri* occurred despite having a fiber concentration which was about one third lower than that for *E. pileatus* (Fig. 2B). No Kruskal–Wallis test was performed because volumetric fiber concentrations were vastly different.

The number of doliolid pellets produced per gonozooid and hour ranged from 4.2 to 6.2. Copepod pellets could not be counted because many of them had been damaged by having been captured by *E. pileatus*.

### Visible Fibers in Pellets

**Fig. 3:**
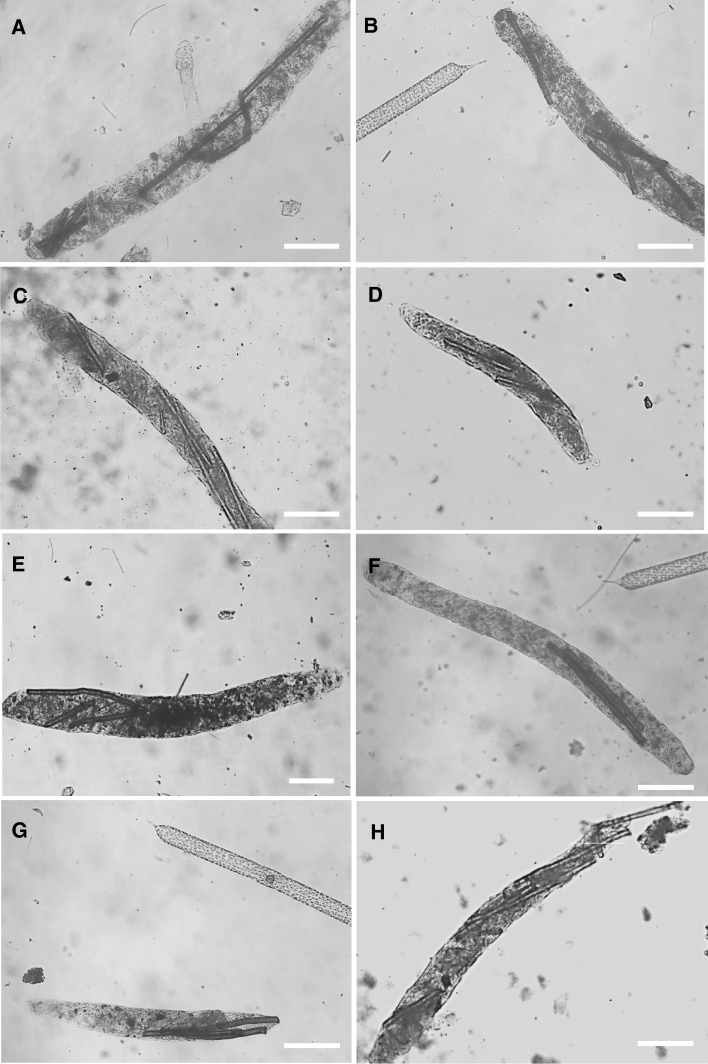
Fecal pellets of *Eucalanus pileatus* having ingested the diatom *Rhizosolenia alata* and nylon fibers of near 10 µm width and about 300 µm length. The scale bar represents 100 µm

Food particles enter the copepod’s gut after usually being broken in the esophagus while they are not broken entering the doliolid’s gut (Paffenhöfer and Köster 2005). We decided to document the contents of fecal pellets of both species. Pellets of *E. pileatus* adult females had an average length of 600 µm and 61 µm width (Fig. 3). The average number of fibers per pellet was 3.5 including some broken ones (Table 2). Any other structures could not been discerned. The copepods had attacked during these experiments many of their pellets and had broken them.

**Table 2:** Fecal pellets produced by *Eucalanus pileatus* females feeding on *Rhizosolenia alata* cells in the presence of nylon microfibers of near 10 µm width and about 300 µm length

Pellet No	Pellet length (mm)	Pellet width (mm)	Pellet volume (mm^3^)	Number of fibers pellet^−1^
A	0.72	0.075	0.0032	6
B	0.63	0.067	0.0022	5
C	0.63	0.067	0.0022	4
D	0.43	0.050	0.0008	2
E	0.62	0.067	0.0022	3
F	0.66	0.057	0.0017	2
G	0.44	0.050	0.0009	2
H	0.63	0.058	0.0017	4
Mean value	0.60	0.061	0.0019	3.50
S.D	0.10	0.008	0.0007	1.41
S.E	0.03	0.003	0.0003	0.50
Range	0.44–0.72	0.050–0.075	0.0008–0.0032	2–6

Pellet numbers refer to microphotographs of pellets in Fig. 3. SD Standard Deviation, SE Standard Error

**Fig. 4:**
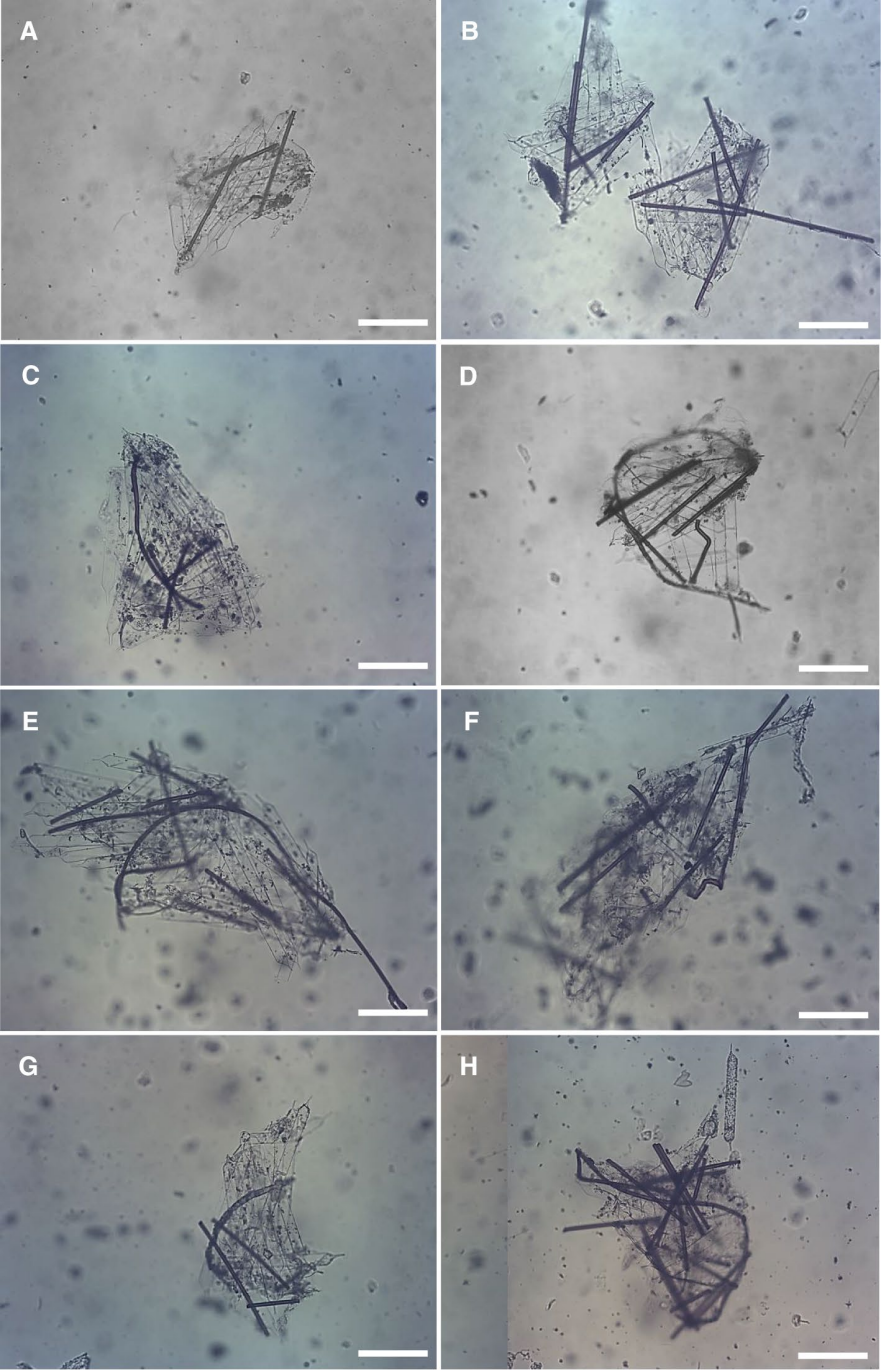
Fecal pellets of gonozooids of *Dolioletta gegenbauri* having ingested the diatom *Rhizosolenia alata* and fibers of 10 µm width and of about 0.3 to 1.45 mm length. The scale bar represents 100 µm

Pellets of *D. gegenbauri* varied widely in dimensions and contents (Fig. 4): Many pellets were largely flat and pillow-like as the food particles are collected by the doliolid on the continuously-produced mucous net which is passed through the esophagus; this is followed by the digestion process and then pellets are released often in the form of a pillow. Of the morphologically diverse pellets produced by the rather small gonozooids of 4.5–4.9 mm length we are presenting a representative group (Table 3, Fig. 4). The number of fibers per pellet ranged from 3 to 16, while the number of *R. alata* cells ranged from 12 to 28 (all largely digested). Several pellets’ fibers included some far longer than the average length of about 300 µm, being as long as 1.45 mm (Fig. 4E), and also being curved (Fig. 4C–E, H). This reveals that the gonozooids feeding process can cover a wide range of particle lengths (without destruction) if the fiber width does not extend much beyond the about 60 µm wide esophagus.

**Table 3:** Fecal pellets produced by *Dolioletta gegenbauri* gonozooids (4.5 to 4.9 mm) feeding on *Rhizosolenia alata* cells in the presence of nylon microfibers of near 10 µm width and about 300 µm length

Pellet No	Pellet area (mm^2^)	Number of fibers pellet^−1^	Number of *R. alata* cells pellet^−1^	Comments
A	0.065	3	12	Most *R. alata* cells aligned
B	0.073	6	14	Right pellet
C	0.136	4	27	Elongated, curved fiber
D	0.115	8	12	Loose pellet, elongated fiber
E	0.234	9	28	Elongated fiber of 1.45 mm length
F	0.160	9	Counting not possible	Cells out of focus
G	0.085	5	18	Most *R. alata* cells aligned
H	0.105	16	Counting not possible	Elongated fiber
Mean value	0.122	7.5	18.5	
S.D	0.052	3.8	6.7	
S.E	0.019	1.4	2.9	
Range	0.065–0.234	3–16	12–28	

Pellet numbers refer to microphotographs of pellets in Fig. 4. SD Standard Deviation, SE Standard Error

## Discussion

### Abundance of Microplastics in the Ocean and in Experiments

In experimental laboratory studies on the effect of microplastics on marine zooplankton the microplastics concentration ranged from 111 to 314 µg dry weight L^−1^ (Table 4). The number of particles ranged from 7 to 100 fibers ml^−1^ and from 37 to 75 beads ml^−1^ (Table 4). In the ocean concentrations of microplastics were about two to three orders of magnitude lower by weight (Lenz et al. 2016, Table 4). These ocean observations were based on different methods of collection and analysis: Desforges et al. (2014) used 64 µm mesh to collect microplastics, and Di Mauro et al. (2017) filtered water collected with Niskin bottles on glass microfiber filters with a retention capacity of 0.7 µm followed by microscopic quantification. The most recent study used the most advanced microscopic technology (Brandon et al. 2020) resulting in the highest abundances of microplastic particles (5 ml^−1^) found so far. We had calculated the dry weights of microplastics from these ocean studies from pictures (Di Mauro et al. 2017) and from two-dimensional values, provided in the papers. The numerical microplastic concentration applied in our present experimental study is numerically at seven fibers ml^−1^ similar to the findings by Brandon et al. (2020) for the California Current at near five fibers ml^−1^; however, the majority of the ocean fiber dimensions are far smaller resulting in dry weight amounts of two orders of magnitude lower.

**Table 4:** Dry weight concentrations of microplastic particles in the ocean and in experiments (experiments from Table 1)

Type of microplastics	Diameter (µm) or dimensions (µm × µm)	Concentration mL^−1^	Dry weight µg L^−1^		References
*Experiments*					
Polystyrene beads	20	75	300		Cole et al. (2015)
Polystyrene beads	20	37	148		Paffenhöfer and Köster (2020)
Fibers	10 × 40	100	314		Coppock et al. (2019)
Fibers	10 × 30	47	111		Cole et al. (2019)
Nylon fibers	10 × 300	5.7–9.0	165		Köster and Paffenhöfer (this paper)
*Ocean*					
Particles	10 × 606	max. 0.009	0.43	Pacific	Desforges et al. (2015)
Fibers	n.d	0.090	0.24	Gulf of Mexico	Di Mauro et al. (2017)
Particles	10	n.d	10^–3^-1	Compilation of field data	Lenz et al. (2016)
Small fibers	2.7 × 60 (from their Fig. 2)	5	1.7	California Current	Brandon et al. (2020)
Large fibers	9.5 × 600	0.01–0.1	0.43–4.3	California Current	Brandon et al. (2020)

### Clearance Rates on Microplastic Particles and Phytoplankton

Whereas the first four papers of Table 4 revealed effects of microplastic on processes of zooplankton, as shown in Table 1, our recent study offering only about seven fibers ml^−1^ showed no effects of those fibers on clearance rates of calanoid copepods and doliolids at environmental abundances of phytoplankton (Fig. 2A). Botterell et al. (2019) in their review found that 45% of the papers showed negative effects of microplastics on zooplankton rates while only 14% showed no effects. Clearance rates of copepods and doliolids of similar weight feeding simultaneously on *Rhizosolenia alata* and fibers of similar length differed. While clearance rates by *E. pileatus* and *D. gegenbauri* on *R. alata* did not differ significantly (*p* > 0.05, Kruskal–Wallis) *D. gegenbauri’s* rates on fibers were twice as high as those of *E. pileatus* (Fig. 2A). This will be discussed in the paragraph on Perception.

Our results from the experiments (*R. alata* in the presence of fibers) and controls (only *R. alata* offered) revealed that clearance rates of *E. pileatus* on *R. alata* did not differ significantly. The same was found for gonozooids of *D. gegenbauri* (Fig. 2A). These findings imply that the fibers at the experimental levels did not affect clearance rates of both zooplankton species.

### Perception of Particles by Calanoid Copepods and Doliolids

Phytoplankton cells were perceived in the calanoid’s feeding current by chemoperception (e.g., Strickler 1982; Paffenhöfer and Lewis 1990). When beads or fibers are offered alone to calanoids they are not or hardly ingested as they do not provide a chemical signal as phytoplankton cells do (Paffenhöfer and van Sant 1985). However, beads were ingested when they arrived simultaneously with diatom cells at the copepod’s mouth (video observations, Paffenhöfer and Van Sant 1985). As that co-occurrence does not happen often the feeding rates on beads were lower than on diatoms: At an abundance of 0.3 mm^3^ L^−1^ of both, *Thalassiosira weissflogii* of 12 µm width and beads of 20 µm diameter, being offered together, *Eucalanus pileatus* copepodid stage V (C V) ingested 2.7 × 10^6^ µm^3^ d^−1^ of beads and 50 × 10^6^ µm^3^ d^−1^ of the diatom. The ratio between ingestion of diatoms and beads was 18.5 to 1. These observations are being supported by Donaghay and Small (1979): when the diatom *T. fluviatilis* was offered together with 20 µm spheres very few spheres were eaten. Huntley et al. (1983) stated that bead consumption was low in all of the experiments when they offered polystyrene beads of 16.5 µm diameter to *Calanus pacificus* copepodid stage IV (C IV). While the diatom *T. weissflogii* was ingested at a clearance rate of 2.5 ml copepodid IV^−1^ h^−1^, the beads were eaten at a rate of only 0.3 ml copepodid IV^−1^ h^−1^. Nauplii of the copepod *Calanus pacificus* did not ingest polystyrene beads when offered together with phytoplankton (Fernandez 1979).

As many calanoids seem to have the ability to perceive food particles by chemosensory (e.g., Paffenhöfer and Loyd 2000, showing chemosensory structures in setae of maxillipeds and second antennae) various particles in the ocean might develop biofilms over days and weeks (e.g., Phuong et al. 2016; Vroom et al. 2017). Polystyrene beads which had been kept in seawater for three weeks were ingested at a higher rate than fresh beads by the calanoid copepods *Calanus finmarchicus* and *Acartia longiremis* (Vroom et al. 2017). The authors assume that over that period a biofilm developed on those beads which provided a chemical signal to those copepods. The possibility of chemical signals triggering the ingestion of microplastics has been supported by two studies: Procter et al. (2019) offered small nylon fibers (10 µm × 30 µm), which had been exposed to DMS (dimethyl sulfide) for six hours, to the calanoid *Calanus helgolandicus.* The copepods ingested significantly more of the DMS-treated-fibers than untreated ones. The results of a similar study by Botterell et al. (2020), also involving the feeding-current-creating *C. helgolandicus,* supported the findings of the previous study.

There are also results which appear not to agree with our earlier presented findings/interpretations: Coppock et al. (2019) offered microplastics at 100 ml^−1^ (fibers or spheres) in 50 ml glass bottles to single *C. helgolandicus* females for 24 h. There was no mention of phytoplankton addition. All pellets revealed numerous microplastics (their Fig. 2) which would mean their ingestion occurred without phytoplankton being present. However, their Fig. 2c, when feeding on fibers, also shows several *Prorocentrum micans* cells in that pellet indicating that phytoplankton was offered with the plastics. This observation leads to the conclusion that phytoplankton could have contributed to the ingestion of fibers in those specific studies.

The feeding of doliolids does not seem to be affected by perception processes as long as the respective particles can enter their mouth (e.g., Deibel 1985, 1998; Köster and Paffenhöfer 2017). That appeared to be the case when we offered 300 µm long fibers and *R. alata*, similar in length to the fibers, simultaneously to gonozooids of *D. gegenbauri*. Yet fibers were cleared at a rate which was about 50% higher than that on diatoms (Fig. 2A). That may occur because the fibers have a smaller diameter than the *R. alata* cells and have no spikes at the end of each cell, making it easier to retain them on the mucous net than the diatom cells; and secondly, quite a few of the *R. alata* cells are dividing or in chains of two cells extending over 500 µm in length, and therefore are not that readily retained on the doliolid’s mucous net.

### Ingestion Rates

Ingestion rates provide the actual amounts of food particles entering a feeder’s gut. Offering the fibers together with *R. alata* cells (~ 10 times the volume of fibers, Fig. 2B) resembles the relatively small numerical amount of fibers in situ, and reflects phytoplankton abundance in neritic intrusion waters (e.g., Yoder et al. 1985). The ingestion rates on fibers are less than 10% of those on *R. alata,* being determined by the size of the ingested particles (Fig. 2B). Not observing any obvious fiber effects on feeding rates in our study might change when phytoplankton abundance and cell size is much lower as observed in oceanic waters like the North Atlantic Subtropical Gyre (NASG, e.g., Paffenhöfer et al. 2003). Then, the volumetric concentration of fibers (Brandon et al. 2020) would be much closer to that of phytoplankton and heterotrophic nanoflagellates (e.g., Sherr and Sherr 2009).

Also the average size of small fibers in Brandon et al. (2020) of ~ 2.7 µm width and ~ 60 µm length represents a volume of 340 µm^3^ which is close to a dinoflagellate of 9 µm diameter, encountered in the NASG. Food perception by calanoids increases with decreasing food concentration (e.g., Paffenhöfer and Lewis 1990). Thus, smaller food sizes of low abundance could still lead to relatively frequent perception, followed by ingestion, probably accompanied by co-ingestion of a similar-sized fiber. This assumption of significant ingestion of nanoplankton cells (2–20 µm diameter) was shown earlier for females of the calanoid *Paracalanus aculeatus* (Paffenhöfer et al. 2003).

### Feeding on Microplastic Particles in the Ocean

Desforges et al. (2014, 2015) found on average 2080 plastic particles m^−3^ (majority ranged from 100–500 µm length) and 28 large calanoid copepods of *Neocalanus cristatus* m^−3^ in the northern Pacific Ocean. That copepod species (Copepodid stage V) cleared 290 ml copepod^−1^ d^−1^ of the diatom *Thalassiosira weissflogii* at 11° C (Frost et al. 1983). Multiplying that rate with the number of *N. cristatus* m^−3^ results in 8120 ml cleared d^−1^ m^−3^. In the Pacific Ocean 8120 ml contain on average 17 microplastic particles of ~ 600 µm average length (Desforges et al. 2014). Feeding at 290 ml copepod^−1^ d^−1^ ten copepods of *N. cristatus* would have encountered in situ 6.0 microplastic particles per day. Desforges et al. (2015) found a total of only 25 plastic particles in the guts of 960 N*. cristatus,* i.e., one particle in 38 copepods. Why had been so few ingested? In the Subarctic Pacific there is a scarcity of larger perceivable food particles, as compared to the US southeastern shelf. This limits the co-occurrence of plastics and food particles in *N. cristatus’* feeding current which would be necessary for the ingestion of a plastic particle (e.g., Paffenhöfer and Van Sant 1985). Therefore, effects of microplastic particles on feeding-current-producing calanoid copepods might be limited in those parts of the ocean where their ingestion depends on the simultaneous occurrence of perceived food particles like phytoplankton cells. While feeding-current producing copepods in the northeast Pacific Ocean do hardly appear to ingest microplastic particles, doliolids like *Dolioletta gegenbauri* could ingest them readily as they are occurring intermittently in abundance on the west coast of the USA and Canada (e.g., Mackas et al. 1991). This would imply that a greater abundance of fibers might affect doliolids more than calanoid copepods.

It appears that fibers at the offered sizes and abundance do not affect the feeding of calanoids and larger doliolids, yet occur in different sizes and shapes of fecal pellets which again will be encountered as potential food particles by different zooplankton taxa.

### Fibers in Fecal Pellets

Evaluating fecal pellets of *Eucalanus pileatus* and *Dolioletta gegenbauri,* we observed major differences. Pellets of the copepod were compact and contained destroyed and digested diatom cells and fibers (Fig. 3). Doliolid pellets contained largely digested but physically undamaged diatom cells and fibers (Fig. 4). Those pellets can be compact, i.e., diatoms close together with few fibers (Fig. 4A, B, C, G), or of similar size but more fibers including long ones (Fig. 4D, E), and also pellets with numerous fibers and closely bunched diatoms (Fig. 4E, F H). While copepods like *E.* *pileatus* will find it difficult to ingest fibers of 1 mm or longer (pers. observation) doliolids appear to experience no difficulties in ingesting such larger fibers. Most of the fibers shown in Di Mauro et al. (2017, their Fig. 4) could be ingested by doliolids but most likely not by calanoid copepods. The doliolid pellets also contain on average more fibers per pellet than pellets produced by calanoids of similar size as doliolid zooids (Figs. 3 vs. 4). Doliolids, of similar weight as copepods, not only have more fibers in their pellets but also produce more pellets per hour (*E. pileatus* ~ 2 pellets h^−1^, *D. gegenbauri* ~ 4 pellets h^−1^). In summary, as doliolids remove far more fibers than copepods they also could be more affected by microplastics than copepods.

In comparison with the copepod pellets the doliolid pellets will sink slowly (Patonai et al. 2011). At a specific weight of very close to 1 g cm^−3^, the fibers should not affect the sinking rates of those pellets that much. These pellets can readily serve as food for other doliolids (Köster and Paffenhöfer 2017) and also copepods as they contain organic matter with a high nutrient value. However, the food value of those doliolid pellets could be reduced in the presence of fibers and negatively affect the ingestion of those pellets.

We might obtain insights on the effects of such microplastic particles, as quantified by Brandon et al. (2020), by offering such in situ particulate matter over hours to days to juveniles and adult stages of the often abundant smaller calanoids like the genera *Clausocalanus* and *Paracalanus*. Particles of near 3 µm width and about 60 µm length, as found by Brandon et al. (2020), could be readily ingested by nauplii and older stages of such genera. That probably would occur only in the presence/co-occurrence of phytoplankton and heterotrophic cells. In the open ocean small phytoplankton (< 10 µm ESD) and heterotrophic cells (< 10 µm ESD) dominate (Paffenhöfer et al. 2003). These food particles are readily perceived and ingested by early copepodid stages of small calanoids (e.g., Berggreen et al. 1988). Calanoid juveniles are more sensitive to food limitations than adults as shown by mortality rates (e.g., Paffenhöfer 1970). Such microplastics might have more likely an effect on those stages if the abundance of the accompanying living food particles is low as found in oceanic waters.

## Data Availability

The data supporting the findings of this study are available within the article.

## References

[CR1] Andrady AL (2011). Microplastics in the marine environment. Mar Poll Bull.

[CR2] Arthur C, Baker J, Bamford H (eds) (2009) Proceedings of the international workshop on the occurrence, effects, and fate of microplastic marine debris. NOAA Technical Memorandum, NOS-OR & R-30, NOAA, Silver Spring, Sept. 9–11, 2008, 530

[CR3] Atkinson LP, Paffenhöfer G-A, Dunstan WM (1978). The chemical and biological effect of a Gulf Stream intrusion off St. Augustine. Florida Bull Mar Sci.

[CR4] Berggreen U, Hansen B, KiØrboe T (1988). Food size spectra, ingestion and growth of the copepod *Acartia tonsa*: implications for the determination of copepod production. Mar Biol.

[CR5] Botterell ZLR, Beaumont N, Dorrington T, Steinke M, Thompson RC, Lindeque PK (2019). Bioavailability and effects of microplastics in marine zooplankton: a review. Environ Poll.

[CR6] Botterell ZLR, Beaumont N, Cole M, Hopkins FE, Strinke M, Thompson RC, Lindeque PK (2020). Bioavailability of microplastics to marine zooplankton: effect of shape and infochemicals. Environ Sci Technol.

[CR7] Bowman TE (1971). The distribution of calanoid copepods off the southeastern United States between Cape Hatteras and southern Florida. Smithson Contrib Zool.

[CR8] Brandon JA, Freibott A, Sala LM (2020). Patterns of suspended and salp-ingested microplastic debris in the North Pacific investigated with epifluorescence microscopy. Limnol Oceanogr Letters.

[CR9] Cole M (2016). A novel method for preparing microplastic fibers. Sci Rep.

[CR10] Cole M, Lindeque P, Halsband C, Galloway TS (2011). Microplastics as contaminants in the marine environment: a review. Mar Poll Bull.

[CR11] Cole M, Lindeque P, Fileman E, Halsband C, Goodhead R, Moger J, Galloway TS (2013). Microplastic ingestion by zooplankton. Environ Sci Technol.

[CR12] Cole M, Lindeque P, Fileman E, Halsband C, Galloway TS (2015). The impact of polystyrene microplastics on feeding, function and fecundity in the marine copepod *Calanus helgolandicus*. Environ Sci Technol.

[CR13] Cole M, Coppock R, Lindeque PK, Altin D, Reed S, Pond DW, Sørensen L, Galloway TS, Booth AM (2019). Effects of nylon microplastic on feeding, lipid accumulation, and moulting in a coldwater copepod. Environ Sci Technol.

[CR14] Conover WJ (1980). Practical nonparametric statistics.

[CR15] Coppock RL, Galloway TS, Cole M, Fileman ES, Queiros AM, Lindeque PA (2019). Microplastics alter feeding selectivity and faecal density in the copepod *Calanus helgolandicus*. Sci Total Environ.

[CR16] Cozar A, Echevarria F, Gonzalez-Cordillo I, Irigoien X, Ubeda B, Hernandez-Leon S, Palma AT, Navarro S, Garcia-de-Lomas J, Ruiz A, Fernandez-de-Puelles ML, Duarte CM (2014). Plastic debris in the open ocean. Proc Nat Acad Sci.

[CR17] Deevey GB (1952). Quantity and composition of the zooplankton of Block Island Sound, 1949. Bull Bingham Oceanogr Collect.

[CR18] Deibel D (1985). Blooms of the pelagic tunicate *Dolioletta gegenbauri*: are they associated with Gulf Stream frontal eddies?. J Mar Res.

[CR19] Deibel D, Bone Q (1998). The abundance, distribution and ecological impact of doliolids. The biology of pelagic tunicates.

[CR20] Desforges J-PW, Galbraith M, Dangerfield N, Ross PS (2014). Widespread distribution of microplastics in subsurface seawater in the NE Pacific Ocean. Mar Poll Bull.

[CR21] Desforges J-PW, Galbraith M, Ross PS (2015). Ingestion of microplastics by zooplankton in the Northeast Pacific Ocean. Arch Env Contam Toxicol.

[CR22] Di Mauro R, Kupchick MJ, Benfield MC (2017). Abundant plankton-sized particles in the shelf waters of the northern Gulf of Mexico. Environ Pollut.

[CR23] Donaghay PL, Small LF (1979). Food selection capabilities of the estuarine copepod *Acartia clausi*. Mar Biol.

[CR24] Fernandez F (1979). Particle selection in the nauplius of *Calanus pacificus*. J Plankton Res.

[CR25] Frost BW (1972). Effects of size and concentration of food particles on the feeding behavior of the marine planktonic copepod *Calanus pacificus*. Limnol Oceanogr.

[CR26] Frost BW, Landry MR, Hassett RP (1983). Feeding behavior of large calanoid copepods *Neocalanus cristatus* and *N. plumchrus* from the subarctic Pacific Ocean. Deep-Sea Res.

[CR27] Fryer G (1986). Structure, function and behavior and the elucidation of evolution in copepods and other crustaceans. Syllogeus.

[CR28] Gibson DM, Paffenhöfer G-A (2000). Feeding and growth rates of the doliolid, *Dolioletta gegenbauri* Uljanin (Tunicata, Thaliacea). J Plankton Res.

[CR29] Huntley ME, Barthel K-G, Star JL (1983). Particle rejection by *Calanus pacificus:* discrimination between similarly sized particles. Mar Biol.

[CR30] Köster M, Paffenhöfer G-A (2017). How efficiently can dolioids (Tunicata, Thaliacea) utilize phytoplankton and their own fecal pellets?. J Plankton Res.

[CR31] Lenz R, Enders K, Nielsen T (2016). Microplastic exposure studies should be environmentally realistic. Proc Nat Acad Sci.

[CR32] Mackas DL, Washburn L, Smith SL (1991). Zooplankton community pattern associated with a California Current filament. J Geophys Res.

[CR33] Monteiro WM, Mureb MA, Valentin J (1975) O plancton na ressurgencia de Cabo Frio (Brasil). IV. Zooplancton. Primeiras consideracoes sobre a composicao dos principais grupos. Publicacao do Instituto de Pesquisas da Marinha*.* 85:1–10?

[CR34] Nakamura Y (1998). Blooms of tunicates *Oikopleura* spp. and *Dolioletta gegenbauri* in the Seto Inland Sea, Japan, during summer. Hydrobiologia.

[CR35] Paffenhöfer G-A, Knowles SC (1979). Ecological implications of fecal pellet size, production and consumption by copepods. J Mar Res.

[CR36] Paffenhöfer G-A, Van Sant KB (1985). The feeding response of a marine planktonic copepod to quantity and quality of particles. Mar Ecol Prog Ser.

[CR37] Paffenhöfer G-A, Lewis KD (1990). Perceptive performance and feeding behavior of calanoid copepods. J Plankton Res.

[CR38] Paffenhöfer G-A, Gibson DM (1999). Determination of generation time and asexual fecundity of doliolids (Tunicata, Thaliacea). J Plankton Res.

[CR39] Paffenhöfer G-A, Köster M (2005). Digestion of diatoms by planktonic copepods and doliolids. Mar Ecol Prog Ser.

[CR40] Paffenhöfer G-A, Köster M (2020). The effects of microplastics on *Dolioletta gegenbauri* (Tunicata, Thaliacea). Arch Environ Contam Toxicol.

[CR41] Paffenhöfer G-A, Loyd PA (2000). Ultrastructure of cephalic appendage setae of marine planktonic copepds. Mar Ecol Prog Ser.

[CR42] Paffenhöfer G-A, Wester BT, Nicholas WD (1984). Zooplankton abundance in relation to state and type of intrusion onto the southeastern United States shelf during summer. J Mar Res.

[CR43] Paffenhöfer G-A, Sherman BK, Lee TN (1987). Summer upwelling of the southeastern continental shelf of the U.S.A during 1981: abundance, distribution and patch formation of zooplankton. Prog Oceanog.

[CR44] Paffenhöfer G-A, Atkinson LP, Lee TN, Verity PG, Bulluck LR (1995). Distribution and abundance of thaliaceans and copepods off the southeastern U.S.A. during winter. Cont Shelf Res.

[CR45] Paffenhöfer G-A, Tzeng M, Hristov R, Smith CL, Mazzocchi MG (2003). Abundance and distribution of nanoplankton in the epipelagic subtropical and tropical open Atlantic Ocean. J Plankton Res.

[CR46] Patonai K, El-Shaffey H, Paffenhöfer G-A (2011). Sinking velocities of fecal pellets of doliolids and calanoid copepods. J Plankton Res.

[CR47] Phuong NN, Zalouk-Vergnoux A, Poirier L, Kamar A, Chatel A, Mouneyrac C, Lagarde F (2016). Is there any consistency between the microplastics found in the field and those used in laboratory experiments?. Environ Poll.

[CR48] Procter J, Hopkins FE, Fileman ES, Lindeque PK (2019). Smells good enough to eat: Dimethyl sulfide (DMS) enhances copepod ingestion of microplastics. Mar Poll Bull.

[CR49] Sherr EB, Sherr BF (2009). Capacity of herbivorous protists to control initiation and development of mass phytoplankton blooms. Aquat Microb Ecol.

[CR50] Strickler JR (1982). Calanoid copepods, feeding currents, and the role of gravity. Science.

[CR51] Takahashi K, Ichikawa T, Fukugama C, Yamane M, Kahehi S, Okazaki Y, Kubota H, Furuya K (2015). *In situ* observations of a doliolid bloom in a warm water filament using a video plankton recorder: bloom development, fate, and effect on biogeochemical cycles and planktonic food webs. Limnol Oceanogr.

[CR52] Tebeau CM, Madin LP (1994). Grazing rates of three life history stages of the doliolid *Dolioletta gegenbauri* Uljanin (Tunicata, Thaliacea). J Plankton Res.

[CR53] Turner JT (2002). Zooplankton fecal pellets, marine snow and sinking phytoplankton blooms. Aquat Microb Ecol.

[CR54] Turner JT (2015). Zooplankton fecal pellets, marine snow, phytodetritus and the ocean’s biological pump. Prog Oceanogr.

[CR55] Valentin JL, Monteiro-Ribas WM (1993). Zooplankton community structure on the east-southeast Brazilian continental shelf. Cont Shelf (18–23°S latitude). Res.

[CR56] Vroom RJE, Koelmans AA, Besseling E, Halsband C (2017). Aging of microplastics promotes their ingestion by marine zooplankton. Environ Pollut.

[CR57] Yoder JA, Atkinson LP, Bishop SS, Blanton JO, Lee TN, Pietrafesa LJ (1985). Phytoplankton dynamics within Gulf Stream intrusions on the southeastern United States continental shelf during summer 1981. Cont Shelf Res.

[CR58] Zar JH (1974). Biostatistical analysis.

